# Shedding Light on Photodynamic Therapy in the Treatment of Necrobiosis Lipoidica: A Multicenter Real-Life Experience

**DOI:** 10.3390/ijms25073608

**Published:** 2024-03-23

**Authors:** Federica Li Pomi, Alfonso Motolese, Alessia Paganelli, Mario Vaccaro, Alberico Motolese, Francesco Borgia

**Affiliations:** 1 Department of Precision Medicine in Medical, Surgical and Critical Care (Me.Pre.C.C.), University of Palermo, 90127 Palermo, Italy; federicalipomi@hotmail.it; 2 Dermatology Unit, Department of Surgery, Infermi Hospital, AUSL Romagna, 47923 Rimini, Italy; alfonsomotolese93@gmail.com; 3 Dermatology Unit, Santa Maria Nuova Hospital IRCCS, 42123 Reggio Emilia, Italy; alessia.paganelli@ausl.re.it (A.P.); alberico.motolese@ausl.re.it (A.M.); 4 Department of Clinical and Experimental Medicine, Section of Dermatology, University of Messina, 98125 Messina, Italy; mario.vaccaro@unime.it

**Keywords:** diabetes, Necrobiosis Lipoidica, pain, PDT, photodynamic therapy, ulcer, wound healing

## Abstract

Necrobiosis Lipoidica (NL) is a dermatological condition characterized by the development of granulomatous inflammation leading to the degeneration of collagen and subsequent formation of yellowish-brown telangiectatic plaques usually localized on the pretibial skin of middle-aged females. Due to its rarity and unclear etiopathogenesis, therapeutic options for NL are not well-standardized. Among them, photodynamic therapy (PDT) is an emerging tool, although its efficacy has primarily been evaluated in single case reports or small case series. This study reports the real-life experience of a cohort of NL patients treated with PDT at the Section of Dermatology of the University Hospital of Messina and Reggio-Emilia. From 2013 to 2023, 17 patients were enrolled —5 males (29%) and 12 females (71%) aged between 16 and 56 years (mean age: 42 ± 13 years), with a median duration of NL of 8 years. The overall complete clearance (>75% lesion reduction) was 29%, while the partial clearance (25–75% lesion reduction) was 59%, with 12% being non-responders. This study adds to the little amount of evidence present in the literature regarding the effectiveness of PDT in the treatment of NL. Variability in treatment responses among patients underscores the need for personalized protocols, optimizing photosensitizers, light sources, and dosimetry. The standardization of treatment protocols and consensus guidelines are essential to ensure reproducibility and comparability across studies.

## 1. Introduction

Necrobiosis Lipoidica (NL) is a dermatological condition characterized by the development of granulomatous inflammation leading to the degeneration of collagen and subsequent formation of yellowish-brown telangiectatic plaques usually localized on the pretibial skin of middle-aged subjects [1]. In the early stages, NL usually starts as red-brown papules and nodules, with violaceous, irregular borders that may be raised and indurated [1]. Over time, the lesions flatten, and a central yellow or orange area becomes atrophic, and, commonly, telangiectasias are visible, taking on the characteristic “glazed-porcelain” sheen. While some individuals may experience pain and itching, most lesions present without symptoms [2]. Numbness of the plaques can also occur. The progression of the condition is typically slow, with spontaneous improvement seen in fewer than 20% of cases. Finally, the plaques usually stabilize, and the emergence of new lesions decreases. Epidemiological data indicate that NL typically manifests in the second–third decades in patients with type 1 diabetes and in the fourth decade in patients with type 2 diabetes and in non-diabetics, with women being three times more likely to be affected than men [3,4]. NL has been found to occur in 0.3–1.2% of patients with diabetes mellitus (DM), but little literature exists on the prevalence of NL in non-diabetic patients [3,5]. The paucity of epidemiological data on NL in other immune-mediated conditions could partly be explained by the absence of adequate clinical screening for this disease, which has traditionally been considered a specific manifestation of DM. Furthermore, most of the cohort studies documented in the literature have focused on therapeutic rather than epidemiological aspects [6]. In this regard, a recent multicenter retrospective study, examining the data of 52 patients with NL of the lower legs, found a prevalence of thyroid function disorders in 13%, compared to 5.5% in the general population, suggesting that NL could be associated with other conditions than DM alone [7]. It was observed that patients diagnosed with both NL and type 1 DM had a higher overall susceptibility to celiac disease in comparison to those with diabetes alone, with the prevalence of celiac disease being 3.4% among patients with both conditions, compared to 1% among patients with type 1 DM [8,9]. In addition to DM, NL patients have also been observed to suffer from dyslipidemia, obesity, hypertension, coronary heart disease, stroke, and reduced renal function, with hypertension being the most frequent secondary diagnosis associated with NL [10]. Moreover, NL has been included in Category III of diseases, which “may occasionally present as Koebner’s phenomenon” occurring after trauma or surgery, according to Weiss et al.’s classification [11]. A single case report also highlighted the onset of NL as Wolf’s isotopic response, namely the occurrence of a new cutaneous disorder at the site of another unrelated and already healed skin disease [11,12]. The pathogenesis of NL remains unknown. Because of its association with DM, some authors hypothesized that NL could be one of the clinical manifestations of microangiopathy; it was initially suggested that hypoxia is part of NL’s pathogenesis due to lower oxygen levels in vessels near NL lesions, which was shown using Doppler analysis [13]. However, Ngo et al. contradicted this finding, revealing higher blood flow in NL lesions compared to unaffected skin [14]. Immunohistochemical analysis of NL lesion tissue supports the hypoxia theory, citing increased glucose transporter 1 receptors in fibroblasts, although this observation is still subject to debate [15]. Notably, the severity of hyperglycemia and the level of diabetic control do not seem to correlate with the presence of NL [16]. Increasing evidence suggests that NL may be induced by immunological mechanisms, in which either an immune complex disease or autoantibodies targeting vessel wall tissue antigens represent the triggering events. This hypothesis is supported by the detection of immunoglobulins and complement factor deposits, especially immunoglobulin M (IgM), C3, and fibrin, in vessel walls and the dermal–epidermal junction of the affected skin [6,17]. An immune-mediated process is also suggested by the stronger association with type 1 DM rather than with type 2 DM. The clinical presentation of NL poses a diagnostic challenge, and therapeutic interventions aim to manage symptoms and halt disease progression. Diagnosis typically involves clinical evaluation and dermoscopic imaging, although biopsy might be necessary to differentiate NL from other similar non-infectious granulomatous conditions, such as granuloma annulare, necrobiotic xanthogranuloma, sclerosing lipogranuloma, cutaneous sarcoidosis, or from infectious granulomatous conditions, including leprosy [5]. Other differential diagnoses include diabetic dermopathy, panniculitis (a group of inflammatory diseases involving subcutaneous fat, including erythema nodosum), morphea, lichen sclerosus, lipodermatosclerosis, and pyoderma gangrenosum.

Histologically, NL has been characterized by horizontally palisaded granulomatous inflammation with intermixed layers of necrobiosis, described as a “sandwich-like” configuration [18]. The disease typically follows a chronic course with a slow and progressive extension of the lesions over years. The risk of ulceration, the limited spontaneous improvement, and the cosmetic worries drive patients to seek medical intervention. Ulceration, often induced by trauma, may occur in up to 30% of patients, sometimes leading to severe pain, with a high risk for secondary infection and considerable impairment of the patient’s quality of life. Due to its rarity and the lack of a full understanding of etiopathogenesis, treatment options have been poorly investigated. Traditional therapies include topical or intralesional corticosteroids, which provide symptomatic relief but may not alter the natural course of the disease [1]. Calcineurin inhibitors and retinoids have been employed as topical alternatives to steroids, with poor results [19]. Systemic therapies, such as immunomodulators and antimalarials, have also been used with varying degrees of success. Some anecdotal reports support the use of tumor necrosis factor-α (TNF-α) inhibitors, topical psoralen and ultraviolet A (PUVA), and fumaric acid esters for treatment [20,21,22,23,24,25,26,27,28,29,30]. However, the quest for more effective and targeted treatments continues to drive research in this field. In this context, photodynamic therapy (PDT) has emerged as a promising modality in the therapeutic armamentarium against NL. PDT represents a non-invasive, light-based treatment strategy that exploits the interaction between a photosensitizing agent, 5-aminolaevulinic acid (ALA) or its methylated ester (MAL), light, and molecular oxygen to induce localized cytotoxicity. This unique approach has been shown to be effective for various dermatological conditions, including both oncological and infectious ones, and its application in granulomatous disease is gaining attention for its potential to address the underlying inflammatory processes [19]. Regarding this topic, PDT has gained a Strength of Recommendation C and Quality of Evidence III, according to the latest guidelines, for the treatment of NL and granuloma annulare, another granulomatous disease in which PDT has been shown to have similar efficacy as other topical treatments [31,32].

Herein, we first report the real-life experience of a cohort of NL patients treated with PDT at the Section of Dermatology of the University Hospital of Messina and Reggio-Emilia. Then, we delve into the current body of evidence supporting the use of PDT in NL treatment, exploring its mechanisms of action, clinical outcomes, and its potential role in the evolving landscape of dermatological interventions. By shedding light on the integration of PDT into the therapeutic spectrum for NL, we aim to contribute to the ongoing discourse on innovative and effective strategies for managing this challenging dermatological condition.

## 2. Results

### 2.1. Patient Characteristics

From 2013 to 2023, 17 patients were enrolled—5 males (29%) and 12 females (71%) aged between 16 and 56 years (mean age: 42 ± 13 years). The median duration of NL was 8 years (±4 years). Ulceration was present in nine patients (53%). All patients had undergone one or more previous treatments: 100% with topical corticosteroids, 88% with calcineurin inhibitors, 41% with intralesional corticosteroids, 29% with occlusive topical corticosteroids, 24% with platelet-rich plasma (PRP), and 5% with pentoxifylline. Six patients underwent an average of six sessions of MAL-PDT at monthly intervals, while 11 patients underwent an average of seven sessions of ALA-PDT every other week. Table 1 summarizes the patients’ characteristics.

### 2.2. Efficacy

**Figure 1:**
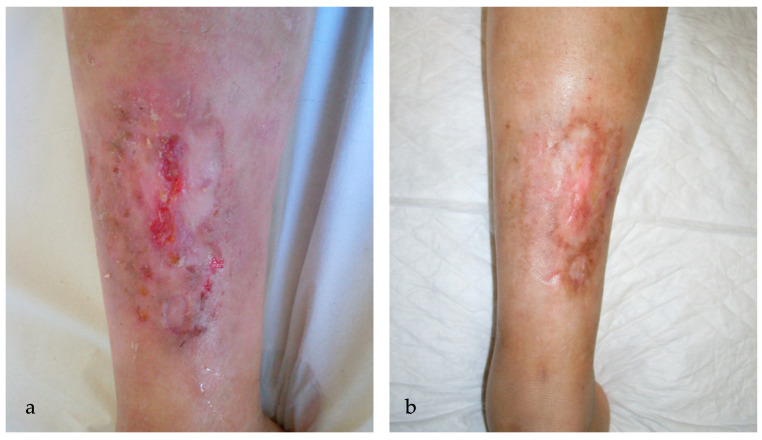
(**a**) An ulcerated plaque (6.5 × 2.7 cm) in the pretibial region of a 39-year-old woman affected by NL for 16 years before starting the therapy with MAL-PDT; during the first session, VAS pain was 9; (**b**) resolution of the pretibial ulceration one month after 5 sessions of MAL-PDT monthly, with VAS pain being 4 during the last treatment.

**Figure 2:**
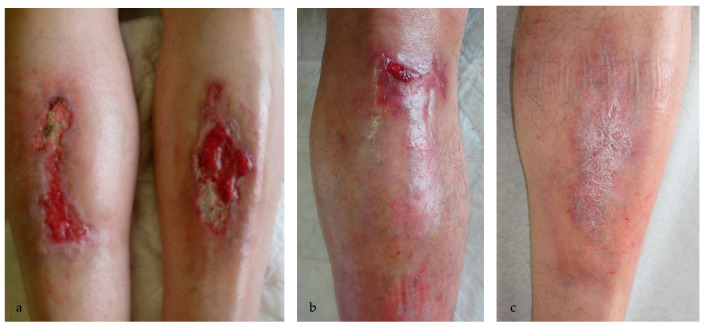
(**a**) Deep ulcerated lesions of the pretibial region (9.5 × 2.5 cm right leg and 7 × 4.2 cm left leg) in a 56-year-old man before starting the therapy with MAL-PDT; (**b**) partial resolution of the ulcer in the left shin; (**c**) complete resolution of the ulcers in the right shin after 6 sessions of MAL-PDT at a one-month interval.

**Figure 3:**
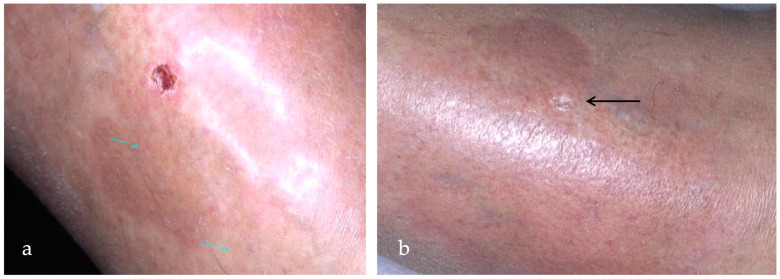
(**a**) An ulcerated NL lesion in the pretibial region of a 53-year-old woman for 6 years before starting the therapy with ALA-PDT every other week; (**b**) complete resolution (>75%) of the ulcerated lesion (black arrow) in the context of erythematous-atrophic plaque after 11 sessions of ALA-PDT every other week.

**Figure 4:**
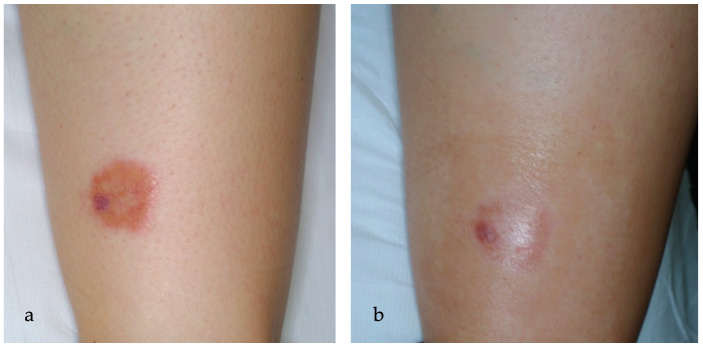
(**a**) An erythematous-orange plaque of the leg in a 16-year-old woman affected by NL for 2 years. In the left part of the plaque, a small, ulcerated lesion is detected, while telangiectasias in the context of granulation tissue are clinically visible; (**b**) partial reduction of the ulcerated lesion after 7 sessions of ALA-PDT every other week.

**Figure 5:**
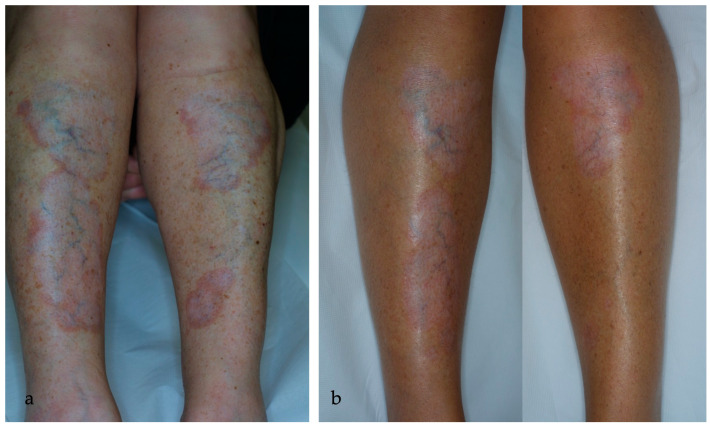
(**a**) NL plaques in the pretibial region bilaterally for 6 years in a 52-year-old woman before therapy; (**b**) partial reduction (75–25%) of the lesions, with lightening of the erythematous rim, and complete resolution of the lesion in the lower left pretibial region after 8 sessions of ALA-PDT.

**Figure 6:**
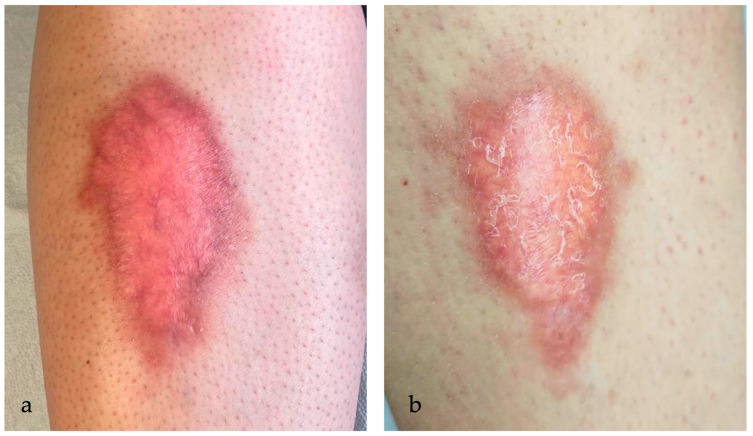
(**a**) A red-brown plaque, with brownish irregular borders raised and indurated in the pretibial region in a 29-year-old woman affected by diabetes and NL for 4 years; (**b**) persistence of the lesion (reduction < 25%) with telangiectasis clinical visible in the context of red-brown plaque after 6 sessions of ALA-PDT every other week.

Complete and partial clearance were achieved in 5/17 (29%) and 10/17 (59%) patients, respectively. Only two patients were classified as non-responders (12%). MAL- and ALA-PDT performed similarly. The MAL-treated group obtained 33% of complete clearance and 67% of partial clearance, while the ALA group obtained 29% of total clearance and 59% of partial clearance. Ulcerative NL showed a better response rate, with 5/9 (55%) lesions achieving complete response and 4/9 (45%) lesions achieving partial response. PDT performed worse with non-ulcerative NL, with partial clearance in 6/8 (75%) patients and no response in 2/8 (25%) patients and without any complete responders. Overall, 75% of non-ulcerative NL patients resulted as responders to PDT whereas 100% of ulcerative NL patients showed a response to PDT. Figure 1, Figure 2, Figure 3, Figure 4, Figure 5 and Figure 6 are examples of complete, partial, and no responses in patients treated with both MAL- and ALA-PDT. Table 2 summarizes the efficacy of PDT.

### 2.3. Visual Analogue Scale (VAS) Pain

Concerning pain, during the first session of PDT, the median VAS pain reported by patients was 8, while the median VAS pain during the last session of PDT was 3. The pain tended to be greater during the first minutes of illumination, with a progressive reduction during the session. The treated areas were cooled using a fan or sprayed with ice water both during and immediately after each session. No patients stopped the treatment for pain or burning sensation during the illumination sessions.

## 3. Discussion

Due to its rarity and unclear etiopathogenesis, therapeutic options for NL are not well-standardized, with no treatments currently approved by the FDA. Typically, initial treatment involves the use of topical or intralesional corticosteroids, which have long been a mainstay in NL management, especially for active and enlarging lesions [1,16]. However, their effectiveness as monotherapy is limited, with less than half of cases experiencing improvement [33]. Furthermore, their use may exacerbate skin atrophy associated with NL, increasing the risk of ulceration and worsening disease morbidity, thus requiring the use of combination therapies. Limited data on the risk of relapse after discontinuing topical corticosteroid treatment precluded any definitive conclusions. In addition to topical corticosteroids, the most supported treatments for NL include topical calcineurin inhibitors, compression therapy, and classic immunosuppressants and immunomodulators such as methotrexate, cyclosporine, and fumaric acid esters [19]. In a review by Nihal et al., patients treated with topical calcineurin inhibitors showed improvement in 11 out of 17 cases, while compression therapy was effective in 15 out of 20 cases [19]. Evidence from retrospective studies suggests limited effectiveness of potassium iodide and dapsone, with improvement seen in two out of seven and three out of seven cases, respectively [34,35], while better performances were recorded with the use of systemic immunosuppressants [19,36,37,38,39]. An improvement in lesion inflammation was reported in a 12-year-old girl after 3 months of oral doxycycline monohydrate (200 mg/die), while another patient did not respond to the therapy [40,41]. Partial healing was observed in two patients after 6 months of doxycycline for the treatment of elbow NL occurring after trauma [42]. Even though the exact mechanism of action in NL is unclear, doxycycline seems to have anti-inflammatory properties and inhibit matrix metalloproteinases (MMPs), which play a role in tissue inflammation [41]. Other treatments, including biologic agents [21,22,23,24,25,26,27,28,29,42,43,44,45,46,47,48,49], Janus Kinase (JAK) inhibitors [50,51,52], and skin grafting [53,54], have shown improvement in individual cases but lack comprehensive data. Specifically, the use of TNF-α inhibitors, including infliximab, etanercept, and adalimumab, is increasingly recognized, with case reports and case series confirming their therapeutical efficacy, especially in ulcerative lesions [21,22,24,25,26,27,28,29,43,48,49]. A recent systematic review reported improvement in 2/2 patients treated with adalimumab, 2/2 treated with infliximab, and 2/2 treated with both adalimumab and infliximab [19]. The pivotal role of TNF-α in granuloma formation provides a theoretical basis for understanding how this class of medications may be effective in managing NL [55]. TNF-α recruits inflammatory cells, including macrophages and T cells, to the site of infection, thus promoting increased macrophage phagocytic activity, which leads to granuloma formation and maintenance. Anti-TNF-α therapies have anti-granuloma effects and are already being used to successfully treat cutaneous non-infectious granulomatous diseases, including sarcoidosis and granuloma annulare [23,56,57,58]. Additionally, recent case reports have demonstrated the effectiveness of ustekinumab, an interleukin (IL)-12/23 monoclonal antibody, in treating NL. Both IL-12 and IL-23 play roles in the formation and persistence of granulomas in infectious and non-infectious conditions. However, the use of ustekinumab for NL is currently limited to small case reports and case series [44,45,46,47]. Regarding the newest JAK inhibitors, Damsky et al. conducted an immunohistochemical analysis revealing increased staining for phosphorylated (p)-STAT1 and (p)-STAT3 in NL lesions, suggesting constitutive activation of JAK/STAT signaling in the disease, providing a potential mechanistic basis for JAK inhibitor use. Additionally, the authors observed synergistic improvement with tofacitinib and intralesional corticosteroids compared to monotherapy, suggesting JAK inhibitors may block JAK-dependent cytokines, while corticosteroids target JAK-independent ones like TNF-α [50]. However, given the little evidence and low quality of data available, it is challenging to reach definitive results on evidence-based therapy recommendations [50,51,52].

Phototherapy has been identified as the most extensively studied and evidence-supported treatment for NL [19]. Nevertheless, due to the various forms of phototherapy available and the numerous factors influencing outcomes, such as treatment frequency and dosage, categorizing this evidence as a uniform class can be challenging. Among the phototherapy methods, PDT, because of its non-invasive nature and minimal systemic side effects, represents an attractive option for patients with NL, especially those seeking alternatives to traditional therapies. PDT is a non-invasive photochemotherapeutic procedure involving the topical application of 5-ALA or its methylated ester, MAL, both being porphyrin-based light-responsive prodrugs, which are rapidly converted by the haem biosynthetic pathway to protoporphyrin IX (PpIX). After a predetermined incubation period and upon irradiation with red light (~630 nm), these prodrugs stimulate cytotoxic reactive oxygen species (ROS) production, selectively destroying cells with a high metabolic state, including infective, tumoral, and inflammatory ones [31]. PDT has been used successfully in the treatment of non-melanoma skin cancer and actinic keratoses, with growing experience in the management of infectious dermatoses; however, less evidence exists for the treatment of inflammatory diseases, including NL [59,60,61,62,63]. The literature shows variable results about the efficacy of PDT, with clearance rates that are often very different from each other, with great variability between the number of sessions and the interval between sessions. The recent evidence regarding PDT is supported by Kaae et al.’s study, which demonstrated an overall cure rate of 66% in patients treated with MAL-PDT, with sustained remission in those who responded positively and minimal risk of relapse [64]. In 2009, Berking et al. conducted a retrospective study on 18 patients, with almost 40% showing some degree of response. However, the authors concluded that PDT could be recommended only in therapy-resistant NL patients but not as first-line therapy [65]. An Italian retrospective analysis conducted on eight patients reported marked efficacy (>75%) in 38% of patients and moderate efficacy (50–75%) in 38% of patients [32]. In a small case series, two out of three patients experienced poor results; however, all patients underwent only a few treatment sessions, thus explaining, at least in part, the limited success of the treatment [66]. Conversely, Bernia et al. reported complete clearance in four female patients after a mean of 3.2 sessions per lesion [67].

Our real-life experience confirms the effectiveness of PDT in necrobiosis, with a total of 88% of patients being responders (29% complete clearance and 59% partial clearance). However, the limits of our study are the small number of patients, which can limit the statistical power and heterogeneity of protocols (since 11 patients received ALA-PDT, while 6 patients received MAL-PDT), as well as the difference in the number of sessions and the interval between sessions. However, from the literature, different protocols emerge among centers and patients belonging to the same center, both related to the number of sessions and the interval between sessions. This is derived from the lack of standardized protocols such that the therapy is mostly based on physicians’ clinical evaluation. Furthermore, in our study, the absence of a control group makes it challenging to directly compare the treated group to another group not receiving treatment. This limitation hampers our ability to definitively attribute the observed outcomes solely to PDT. Finally, the subjectivity of the therapeutic success assessment, depending on the dermatologists’ visual clinical evaluation, can generate inter-individual variability bias. Limitations arise from clinical presentations which may vary and, at the same time, result from the lack of a comprehensive cross-study definition of what constitutes successful treatment. However, to the best of our knowledge, no other standardized clinical evaluation tools are available to date.

The application of PDT in NL is thought to induce local immunomodulation, reduce inflammation, and promote tissue repair. In NL, this targeted approach aims to disrupt the abnormal vascularization and inflammatory cascade underlying lesion formation. In addition to its broader anti-inflammatory properties, PDT appears to have a beneficial impact on NL by altering the collagen matrix, thereby promoting wound healing and ameliorating sclerosis [68]. PDT promotes the production of MMP in fibroblasts, leading to enhanced levels of MMP-1, MMP-9, and transforming growth factor (TGF)-β3 in treated wounds compared to untreated wounds [69,70]. Moreover, building upon the study by Kim et al. [71], who successfully treated granuloma annulare—a granulomatous condition sharing similarities with NL—with PDT, it can be hypothesized that PDT may also be effective in NL through the accumulation of the photosensitizer in lymphatic infiltrates and inhibition of T-cell proliferation [72]. Regarding this topic, disorders with chronic granulomatous inflammation and/or degenerative changes of collagen have been demonstrated to be highly responsive to PDT and very well tolerated with low pain and little local inflammation [32]. It has been hypothesized that the overlying normal or slightly atrophic epidermis hindered the penetration of photosensitizer, thus leading to low skin sensitization and low pain, allowing for the modulation of the dermal granulomatous processes [73]. PDT has also been shown to be effective in wound healing and the treatment of skin ulcers, which characterize NL lesions. Our data confirm the good performance of PDT in ulcerated NL lesions and wound healing, with ulcerative lesions responding better than non-ulcerative ones (100% vs. 75% response rate, respectively). The process of wound healing involves a plethora of cellular, molecular, and biochemical events that culminate in the restoration of damaged tissues [74]. Initially, when a lesion occurs, mediators are released, starting the repair process by triggering inflammation and the migration of leukocytes and platelets. This is followed by a proliferative stage characterized by reepithelialization, angiogenesis, and an increase in fibroblast concentration. Subsequently, remodeling occurs, enhancing collagen fiber deposition and promoting water reabsorption to strengthen scars and reduce their thickness [75]. Acute inflammation occurs immediately in response to a damaging agent and, if promptly resolved, is typically beneficial and self-limiting. Conversely, chronic inflammation persists even after the removal of the initiating agent, often involving monocytes, macrophages, and lymphocytes. While acute inflammation promotes wound healing, chronic inflammation hinders the healing process. Regarding this topic, PDT has emerged as a potential intervention by promoting acute inflammation, thereby altering the physiological course of chronic wounds, whether infected or not, and facilitating healing. PDT has been suggested to induce a localized acute inflammatory response, activating the immune system, with neutrophils being the first immune cells recruited to the inflammation site, facilitated by TNF-α production. Subsequently, myeloid cells, monocytes, macrophages, and mast cells accumulate, leading to the activation of T CD8+ cells, which eliminate damaged cells and tissues [76]. PDT also influences neutrophil activation, contributing to increased production of pro-inflammatory cytokines. Concurrently, during the resolution of acute inflammation and restoration of tissue homeostasis, lipid mediators are produced, exerting anti-inflammatory and immune-modulatory effects. These include inhibiting leukocyte chemotaxis and blocking TNF-α and IL-6 production while promoting IL-10 expression [74]. To ensure proper wound remodeling, a balance between extracellular matrix synthesis and degradation is crucial. PDT modulates the production of TGF-β, favoring controlled collagen fiber deposition. Moreover, MMPs play a role in tissue remodeling by regulating collagen degradation and extracellular matrix remodeling, with various cell types expressing MMPs, including keratinocytes, fibroblasts, endothelial cells, and inflammatory cells [74].

**Figure 7:**
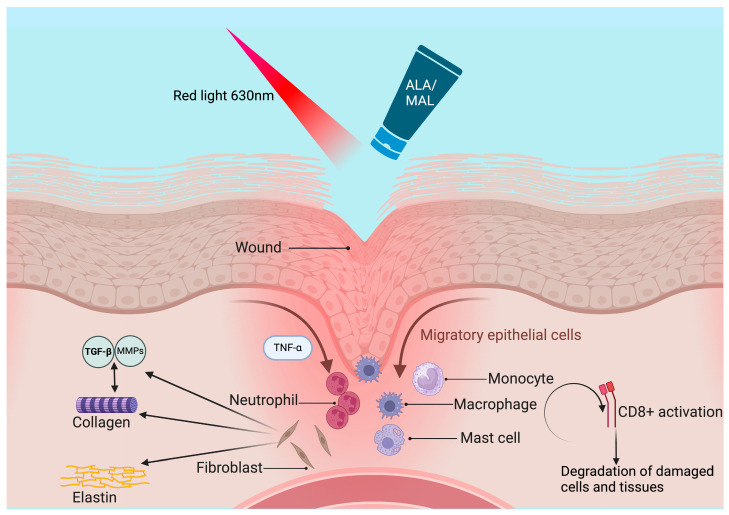
PDT is thought to induce a localized acute inflammatory response, activating the immune system, with neutrophils being the first immune cells recruited to the inflammation site, facilitated by TNF-α production. Subsequently, monocytes, macrophages, and mast cells accumulate, leading to the activation of T CD8+ cells, which eliminate damaged cells and tissues. PDT also influences neutrophil activation, with subsequent increased production of pro-inflammatory cytokines. PDT modulates the production of TGF-β, favoring collagen fiber deposition. Moreover, MMPs play a role in tissue remodeling by regulating collagen degradation and extracellular matrix remodeling. Created with BioRender.com.

The good effectiveness of PDT in the treatment of ulcerative NL, performing better than in non-ulcerative lesions, as evidenced in our study, may suggest the use of this method, especially in ulcerative lesions, by virtue of its proven wound-healing capacity. However, the lack of response may be due to different factors. One constraint of using PDT for NL may arise from the dermal and subcutaneous localization of the disease, which can impair the accumulation and activation of the photosensitizers. It is well-known, in fact, that PDT performs better within a depth of 2 mm. Additionally, inadequate penetration into altered tissue or the granulomatous nature of the disease may hinder the cellular uptake of the photosensitizer, serving as another potential impediment to PDT efficacy. Another limiting factor of PDT for NL could be pain, especially in ulcerated lesions, as it represents the most frequent and limiting side effect [32]. The painful burning sensation usually begins immediately or very early during light exposure, quickly becoming very intense, peaking in the first few minutes of treatment. Subsequently, the pain usually tends to decrease or even ease towards the end of the treatment. ROS seems to play a central role in generating pain sensations by activating sensory neurons that transmit the signals to the brain’s sensory cortex. Pain intensity seems to be correlated with the depth of singlet oxygen production in the skin, which is in turn influenced by the properties of the photosensitizer and the wavelength of the light used for stimulation. Localized hypoxia resulting from oxygen-consuming reactions can lead to a decrease in tissue pH, thus triggering pain signals due to reduced oxygen levels surrounding mitochondria-rich nerves [77]. Nevertheless, the effectiveness of PDT does not seem to correlate with the intensity of the local inflammatory reaction, which, instead, seems to be generally proportional to the extent of pain and burning felt by the patient. The main bias about pain is the subjective evaluation of this individual sensation, with large inter-patient variability. The most common score method is the VAS scale, which is, unfortunately, arbitrary and does not have reproducible results. Although pain during illumination sessions is very frequent, in our study, no patient discontinued the therapy due to the onset of the symptom. This is in line with Berking et al.’s retrospective study, where only 1 in 18 patients stopped the treatment due to the onset of pain, with a VAS scale ranging from 2 to 10 (with a median of 5) [65]. Furthermore, Kaae et al., in their study on 65 patients, suggested the use of daylight PDT, based on sunlight exposure, to reduce the intensity of pain, which is probably due to the continuous production and photoactivation of small amounts of PpIX, with decreased local concentration of ROS and, consequently, reduced stimulation of nerve endings [64,77]. Figure 7 represents the main mechanisms of PDT in wound healing. Table 3 and Table 4 summarize the case reports, case series, and retrospective multicenter studies about the use of PDT for NL reported in the literature so far.

## 4. Materials and Methods

### 4.1. Inclusion and Exclusion Criteria

This study was designed as an observational descriptive retrospective study to evaluate the efficacy of MAL- and ALA-PDT in the treatment of NL. This study was conducted in compliance with Good Clinical Practice and local regulatory requirements. Written informed consent was obtained from each participant before enrolment. All patients, referred to the dermatologic units from 2013 to 2023 and treated with MAL- and ALA-PDT at collaborating centers, were selected from complete lists of PDT treatments performed in the centers and retrospectively evaluated to check for the eligibility criteria. Only patients in which conventional topical or systemic therapies were not effective, discontinued because of the development of adverse events, or contra-indicated because of co-morbidities, concurrent therapies, or a high hazard of toxicity were enrolled. The exclusion criteria were pregnancy or lactation, any active systemic infectious disease, other inflammatory, infectious, or neoplastic skin diseases in the affected area, allergy to MAL, ALA, or ingredients in the cream, a history of photosensitivity, the use of immunosuppressive or photosensitizing drugs, and a history or indicators of poor compliance.

### 4.2. Study Design

#### Baseline Evaluation

Data including age, gender, duration of NL, ulceration, personal history of diabetes, and previous topical, systemic, and physical treatments were collected. The diagnosis was assessed clinically in most patients, and a biopsy for histological confirmation was taken only in selected cases. Clinical pictures were recorded.

### 4.3. Treatment Received by Patients

All patients received PDT. The number and frequency of treatments were chosen by the investigators according to their experience and patients’ needs. PDT was initiated by the topical application of a photosensitizer onto the lesions. Two protocols were performed. We used MAL cream (Metvix^®^ 160 mg/g) in 6 patients and 10% ALA (Biosynth AG, Staad, Switzerland) in polyethylene glycol ointment in 11 patients. The lesions were then covered with an occlusive and light-protective dressing for 3 h. After removal of the dressing, the MAL-treated areas were illuminated with red light at a dose of 37 J/cm^2^ (Aktilite lamp, Photocure ASA, Oslo, Norway), while the ALA-treated areas received a dose of 75 J/cm^2^ (S630, AlphaStrumenti, Milan, Italy). To relieve pain or a burning sensation experienced during illumination, the treated areas were cooled using a fan or sprayed with ice water both during and immediately after the session. Cool packs were also frequently applied. Topical or regional anesthesia was not employed.

### 4.4. VAS Pain

VAS pain, consisting of a 10-cm line, with two endpoints representing 0 (‘no pain’) and 10 (“pain as bad as it could possibly be”), was used to assess patients’ pain intensity during the first and last sessions of PDT.

### 4.5. Evaluation Post-Treatment

Clinical pictures were recorded one month after the last session to evaluate treatment efficacy. Efficacy was evaluated as total clearance (>75% reduction of lesions), partial clearance (25–75% reduction of lesions), and no response (<25% reduction of lesions).

### 4.6. Objectives and Endpoints

This study aimed to evaluate the clinical efficacy of PDT for NL treatment and the potential differences in therapeutic response with respect to MAL-PDT and ALA-PDT. The primary efficacy outcome was the percentage of lesions with complete (>75%) clearance, while the second efficacy outcome was the percentage of lesions with partial clearance, defined as a reduction between 75% and 25% of clinical lesions, for both ALA- and MAL-PDT. Specifically, complete clearance was defined as the resolution of erythema and complete healing of the ulceration, if present, with possible scar formation or hypo- or hyperpigmented post-inflammatory patches, observed at the end of the treatment period. Lesions characterized only by a partial reduction of the erythematous plaque, with the persistence of atrophy, were considered partial responses. The secondary objectives were to evaluate the difference in response to PDT between NL ulcerative and non-ulcerative lesions.

### 4.7. Statistical Analysis

Results were expressed as mean ± standard deviation (continuous variables) or absolute frequency and percentage (categorical variables).

## 5. Conclusions and Future Perspectives

Our real-life experience confirms PDT as a reliable tool to treat a chronic, treatment-resistant disease like NL, especially for ulcerative lesions, representing a valid alternative to traditional topical therapies or to innovative but expensive biological drugs. The minimally invasive approach with favorable clinical outcomes and the high safety profile are its major strengths. As our understanding of NL pathogenesis deepens, novel approaches integrating PDT with complementary modalities such as immunomodulators or targeted therapies could enhance treatment outcomes in the near future. Furthermore, advances in photosensitizer development and drug delivery technologies hold promise for refining PDT’s efficacy and safety profile in NL management. Collaborative efforts involving dermatologists, photobiologists, and biomedical engineers are crucial to advancing PDT as a mainstream therapeutic option for NL and expanding its applicability to other dermatological conditions. However, despite its promises, PDT in NL therapy faces certain challenges. Variability in treatment response among patients underscores the need for personalized protocols, optimizing photosensitizer type, light source, and dosimetry. Additionally, the cost-effectiveness of PDT relative to conventional therapies warrants further investigation, particularly concerning its long-term outcomes and healthcare resource utilization. Moreover, the standardization of treatment protocols and consensus guidelines are essential to ensure reproducibility and comparability across studies.

## Figures and Tables

**Table 1:** Patients’ characteristics. TS: corticosteroids; OTS: occlusive topical corticosteroids; ILS: intralesional corticosteroids; CI: calcineurin inhibitors; PRP: platelet-rich plasma; CR: complete response; PR: partial response; NR: no response.

Patient	Sex	Age	NL Duration	Ulceration	Diabetes	Previous Therapy	PDT	N° Sessions, Interval	Outcome
1	F	56	10 years	No	No	TS; ILS; CI; PRP	MAL	7, monthly	PR
2	F	39	16 years	Yes	Yes	TS; ILS; CI; PRP	MAL	5, monthly	CR
3	M	56	12 years	Yes	Yes	TS; ILS; CI; PRP	MAL	6, monthly	PR
4	M	56	12 years	Yes	Yes	TS; ILS; CI; PRP	MAL	6 monthly	CR
5	F	26	16 years	No	Yes	TS; ILS; CI; penthoxifilline	MAL	7, monthly	PR
6	M	29	5 years	No	Yes	TS; ILS	MAL	5, monthly	PR
7	F	44	7 years	Yes	Yes	TS; ILS; OTS	ALA	5, two weeks	CR
8	F	52	6 years	No	Yes	TS; CI	ALA	8, two weeks	PR
9	F	16	2 years	Yes	No	TS; CI; OTS	ALA	7, two weeks	PR
10	F	53	6 years	Yes	Yes	TS; CI	ALA	11, two weeks	CR
11	F	24	3 years	Yes	No	TS; CI	ALA	6, two weeks	PR
12	F	29	4 years	No	Yes	TS; CI, OTS	ALA	6, two weeks	NR
13	F	36	8 years	Yes	No	TS; CI	ALA	5, two weeks	CR
14	M	56	12 years	No	Yes	TS; CI; OTS	ALA	7, two weeks	PR
15	F	35	8 years	Yes	Yes	TS; CI	ALA	8, two weeks	PR
16	M	49	5 years	No	Yes	TS; CI, OTS	ALA	8, two weeks	PR
17	F	39	12 years	No	No	TS; CI	ALA	6, two weeks	NR

**Table 2:** Clearance rates of MAL- and ALA-PDT.

Clearance	MAL-PDT	ALA-PDT	MAL+ALA
Complete clearance (>75%)	2 (33%)	3 (27%)	5/17 (29%)
Partial clearance (25–75%)	4 (67%)	6 (55%)	10/17 (59%)
Non-responders (<25%)	0 (0%)	2 (18%)	2/17 (12%)

**Table 3:** Case reports and case series reported so far in the literature are summarized. TS: corticosteroids; CI: calcineurin inhibitors; ASA: acid acetylsalicilic; UVB: ultraviolet B; PDL: pulsed-dye-laser; CR: complete response; PR: partial response; NR: no response.

Author	Age, Sex	Ulceration	Diabetes	Previous Therapies	PDT	N° Sessions, Interval	Outcome
Lopez Sanz et al. [78]	18, F	No	Yes	TS, CI, oral cyclosporine, PUVA phototherapy	Daylight ALA-PDT	2, bi-monthly	CR
Bernia et al. [67]	21, F	No	Yes	Penthoxyfilline, TS, CI, ASA, calcitriol, laser PDL	MAL-PDT	11, two week	CR
	32, F	No	Yes	Pentoxifillina, TS	MAL-PDT	18, two week	CR
	22, F	No	Yes	TS, CI, PDL	MAL-PDT + ALA	10, two week	CR
	61, F	No	Yes	TS, cryotherapy	MAL-PDT + ALA	6, two week	CR
Borgia et al. [6]	44, F	No	Yes	Not reported.	10% ALA-PDT	6, monthly	PR
Borgia et al. [68]	44, F	Yes	Yes	TS, CI, UVB phototherapy	10% ALA-PDT	6, two week	CR
Kosaka et al. [79]	66, F	No	Yes	Not reported	20% ALA-PDT	9, 2–3 weeks	CR
De Giorgi et al. [80]	31, F	No	Yes	Not reported	10% ALA-PDT	4, monthly	CR
Heidenheim et al. [81]	60, F	No	Yes	TS, systemic ascorbic acid and vitamin E, cryotherapy, radiotherapy using grenz rays, systemic allopurinol	MAL-PDT	3, every week	CR
Truchelo et al. [66]	60, F	No	Yes	TS	MAL-PDT	2	NR
	35, F	Yes	No	TS	MAL-PDT	1	NR
	28, F	No	Yes	TS	MAL-PDT	3	PR

**Table 4:** The retrospective studies reported so far in the literature are summarized below. CR: complete response; PR: partial response.

Authors	Type of Study	PDT	Study Characteristics
Berking et al. [65]	Retrospective multicenter	MAL- and ALA-PDT	18 patients (aged 16–62 years) from 3 European departments were treated with MAL- or ALA-PDT. CR was seen in 1/18 patients after 9 PDT cycles, and PR was seen in 6/18 patients (2–14 PDT cycles), with an overall response rate of 39% (7/18).
Kaae et al. [64]	Retrospective	Conventional and daylight MAL-PDT	80 treatments (70 conventional and 10 daylight PDT) were performed on 65 NL patients. Conventional MAL-PDT had a 100% cure rate of 64% (45/70), while daylight PDT had a 100% cure rate in 80% of the treatment series (8/10), with an overall cure rate of 66% (53/80).
Calzavara-Pinton et al. [32]	Retrospective multicenter	MAL-PDT	8 female patients aged 35 ± 16.9 underwent 10.0 ± 7.5 treatments with an interval of 18.0 ± 12.0 days. A total of 3/8 patients had a marked improvement (>75% lesion reduction) after PDT, 3/8 had moderate improvement (50–75% lesion reduction), and 2/8 reported poor results.

## Data Availability

Data are contained within the article.
